# Comparison of the efficacy of noninvasive positive pressure ventilation combined with and without budesonide/glycopyrrolate/formoterol fumarate metered dose inhaler in the treatment of acute exacerbations of Chronic Obstructive Pulmonary Disease

**DOI:** 10.12669/pjms.41.4.11507

**Published:** 2025-04

**Authors:** Jielu Sun, Xinkai Xu, Fang Wang

**Affiliations:** 1 Jielu Sun, Department of Respiratory and Critical Care Medicine, First People’s Hospital of Yongkang City, 599 Jinshan West Road, Yongkang, Zhejiang Province 321300, P.R. China; 2 Xinkai Xu, Department of Respiratory and Critical Care Medicine, First People’s Hospital of Yongkang City, 599 Jinshan West Road, Yongkang, Zhejiang Province 321300, P.R. China; 3 Fang Wang, Department of Respiratory and Critical Care Medicine, First People’s Hospital of Yongkang City, 599 Jinshan West Road, Yongkang, Zhejiang Province 321300, P.R. China

**Keywords:** Acute exacerbations, Budesonide/glicopyrrolate/formoterol fumarate, Chronic obstructive pulmonary disease, Metered dose inhibitor, Noninvasive positive pressure ventilation

## Abstract

**Objective:**

Mechanical ventilation, such as noninvasive positive pressure ventilation (NIPPV), is often used to relieve the symptoms of acute exacerbations of chronic obstructive pulmonary disease (AECOPD), but its treatment effect is limited. This study explored the application effect of NIPPV combined with budesonide/glycopyrrolate/formoterol fumarate metered dose inhaler (BGF MDI) in AECOPD.

**Methods:**

The clinical records of 152 patients with AECOPD treated in the First People’s Hospital of Yongkang City from March 2023 to March 2024 were retrospectively selected and grouped based on the ventilation method used: simple NIPPV treatment (n=78) and NIPPV combined with BGF MDI (n=74). The primary outcome was improvement in oxygenation; the secondary outcomes were the recovery of body function and levels of inflammatory markers.

**Results:**

After two weeks of treatment, the arterial partial pressure of carbon dioxide (PaCO2) in the combination group was significantly lower than in the NIPPV group, while the arterial partial pressure of oxygen (PaO2) and oxygenation index (OI) were significantly higher (P<0.05). The COPD Assessment Test (CAT) and St George’s Respiratory Questionnaire (SGRQ) scores of the combined group were significantly lower, while the 6-minute walking test (6MWT) score was higher compared to the NIPPV group (P<0.05). The levels of C-reactive protein (CRP) and interleukin-6 (IL-6) in the combined group were significantly lower compared to the NIPPV group (P<0.05).

**Conclusions:**

NIPPV combined with BGF MDI has higher benefits than NIPPV alone for treating patients with AECOPD.

## INTRODUCTION

Chronic obstructive pulmonary disease (COPD) is a common respiratory disease that mainly manifests as ventilation dysfunction and is often accompanied by varying degrees of respiratory distress.^1^ During the acute exacerbation period, COPD may lead to breathing difficulties and rapid progression of the disease.^1^,^2^ In the absence of timely and effective intervention, respiratory failure may occur, affecting the disease outcomes.^1^ȓ^3^ Therefore, early implementation of safe and effective treatment for acute exacerbations of COPD (AECOPD) is of great significance.^4^

Currently, interventions for COPD are often implemented through correcting electrolyte imbalances and anti-infection measures, which can alleviate patients’ clinical symptoms. However, the overall effect often does not meet clinical expectations.^1^,^4^ Noninvasive positive pressure ventilation (NIPPV), which is commonly used in AECOPD as a non-invasive ventilation method, was shown to improve patient comfort and prevent stress reactions caused by invasive ventilation.^5^,^6^ However, the treatment effect of NIPPV is limited for some patients.^6^ Budesonide/glycopyrrolate/formoterol fumarate metered dose inhaler (BGF MDI) is also routinely used for the clinical treatment of COPD,^7^ and exerts its effect through several different pathways. Glycopyrrolate can inhibit the M3 acetylcholine receptor, thereby expanding the airway; formoterol fumarate can relieve asthma and dilate the bronchi, and budesonide can exert anti-inflammatory effects.^8^

Several studies have shown that BGF MDI has achieved high benefits in treating COPD.^7^,^8^ However, the research on the application value of NIPPV combined with BGF MDI in patients with AECOPD is scarce. This study compared the efficacy of NIPPV combined with BGF MDI in treating AECOPD. Our results may help to optimize the treatment of patients with AECOPD.

## METHODS

This retrospective single-center study included records of patients with AECOPD treated at the First People’s Hospital of Yongkang City between March 2023 and March 2024.

### Ethical Approval:

The ethics committee of our hospital approved this study with the number: YKSDYRMYYEC2024-LW-KS-021-01, Date: 2024-06-28.

### Inclusion criteria:

- Patients met the diagnostic criteria for AECOPD.^1^
- Age above 40 years.
- Complete clinical data.

### Exclusion criteria:

- Patients with severe renal, hepatic and cardiac dysfunction.
- Patients with malignant tumors.
- Patients with infectious diseases.
- Patients with hematological and endocrine system diseases.
- Patients with pneumonia, bronchial asthma, and other respiratory diseases.

### Basic treatment:

Upon admission, routine symptomatic treatments such as bronchodilators, anti-infective agents, glucocorticoids, and expectorants were given to all patients. Patients in the NIPPV group were given NIPPV alone, while the Combined group was given BGF MDI treatment before receiving NIPPV, and NIPPV was administered after adequate expectoration.

### NIPPV:

The NIPPV was administered using the Weikang Vivion ventilator. Dual level positive airway pressure ventilation was selected and adjusted to S/T mode. The initial expiratory pressure was set to 4-5 cmH2O, respiratory rate to 12-14 times/min, and inspiratory pressure to 8-10 cmH2O for 20 minutes. The respiratory pressure was adjusted to 5-6 cmH2O and inspiratory pressure to 15 cmH2O based on the patient’s condition and tolerance, while maintaining a blood oxygen saturation of 85% -95%.

### BGF MDI treatment:

BGF MDI, manufactured by AstraZeneca Dunkerque Production, France, was selected. Specification: 120 puffs per bottle (each puff contains 160 μ g of budesonide+7.2 μ g of glycopyrrolate +4.8 μ g of formoterol fumarate), two puffs per serving, twice a day for two weeks.

The following indicators of patients were collected before and two weeks after treatment:

- Oxygenation. Partial pressure of oxygen (**PaO2**), partial pressure of carbon dioxide (**PaCO2**), and oxygenation index (OI)^9^ were measured using the automatic blood gas analyzer.
- Rehabilitation status of bodily functions, including clinical symptom rehabilitation status, respiratory condition, and exercise ability. The rehabilitation of clinical symptoms was evaluated using the COPD assessment test (CAT),^10^ with a total of 40 points. Lower CAT score indicates milder symptoms. The St. George’s Respiratory Questionnaire (SGRQ)^11^ was used to assess respiratory status, with a total of 100 points. Lower score indicates better respiratory status. Exercise ability was assessed based on the six-minute walk test (6MWT).^12^
- Serum levels of inflammatory markers, including C-reactive protein (CRP) and interleukin-6 (IL-6) were detected by enzyme-linked immunosorbent assay using the reagent kit from Wuhan Doctoral Biotechnology Co., Ltd.

### Statistical Analysis:

All data analyses were conducted using SPSS 26.0 software (IBM Corp, Armonk, NY, USA). The Shapiro Wilk test was used to evaluate the normality of the evaluation data. Normal distribution data were represented by mean ± standard deviation (SD), independent sample t-test was used for inter-group comparison, and paired t-test was used for intra-group comparison before and after the intervention. Non-normally distributed data were represented by median and interquartile ranges. The Mann-Whitney U test was used for inter-group comparisons, and the Wilcoxon signed-rank test was used for intra-group comparisons. The count data were represented by the number of cases using the chi-square test. P<0.05 was considered statistically significant.

## RESULTS

**Fig.1:**
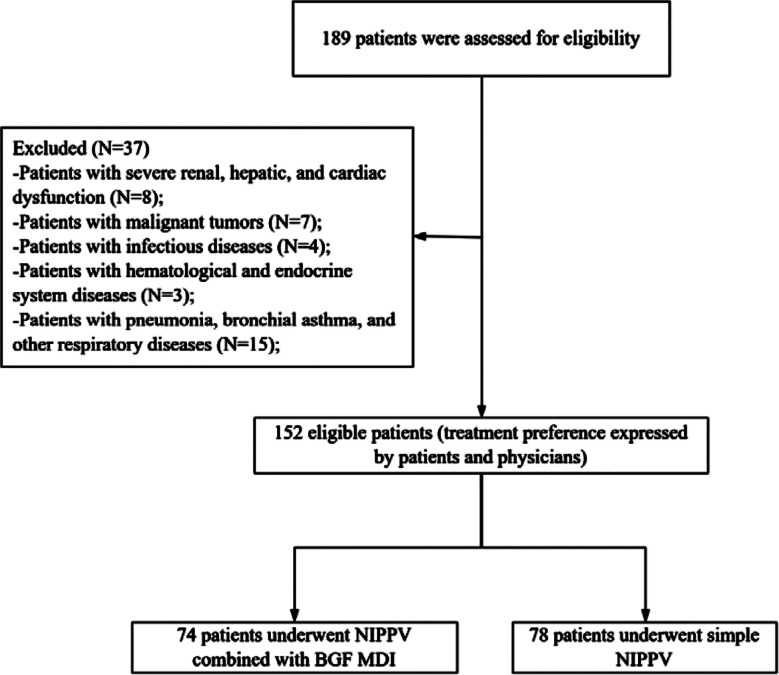
Patient screening process diagram; NIPPV: Noninvasive positive pressure ventilation; BGF MDI: Budesonide/Glycopyrrolate/formoterol fumarate.

This study included a total of 152 COPD patients (75 males and 77 females), with the ages ranging between 45 and 82 years (average age 61.54 ± 7.75 years). Based on the treatment, 74 patients who received NIPPV combined with BGF MDI treatment comprised the combination group, and 78 patients treated by NIPPV alone were retrospectively assigned to the NIPPV group. The patient screening process is shown in Fig.1. There was no significant difference in clinical characteristics between the two groups of patients (P>0.05) (Table-I).

**Table-I:** Comparison of clinical characteristics between two groups.

Characteristics	Combined group (n=74)	NIPPV group (n=78)	χ^2^/t/Z	P
Male (yes), n (%)	42 (56.8)	33 (42.3)	3.172	0.075
Age (years), mean ± SD	60.64±6.84	62.40±8.48	-1.405	0.162
Respiratory rate (times/minute), M(P25/P75)	21 (19-24)	20 (18-23)	-1.640	0.101
Heart rate (beats/minute), M(P25/P75)	79.5 (72-84)	77.5 (69-85)	-0.513	0.608
Disease duration (years), M(P25/P75)	6 (5-8)	7 (5-8)	-1.537	0.124
Drinking alcohol (yes), n (%)	44 (59.5)	41 (52.6)	0.732	0.392
Smoking (yes), n (%)	48 (64.9)	45 (57.7)	0.823	0.364
Coronary heart disease (yes), n (%)	7 (9.5)	8 (10.3)	0.027	0.869
Diabetes (yes), n (%)	11 (14.9)	15 (19.2)	0.510	0.475
Hypertension (yes), n (%)	21 (28.4)	16 (20.5)	1.276	0.259

Before treatment, there was no significant difference in the levels of PaCO2, PaO2, and OI between the two groups (P>0.05). Both intervention methods were associated with a significant decrease in the post-treatment PaCO2, while post-treatment PaO2 and OI increased in both groups compared to pre-treatment values (P<0.05). Post-treatment PaCO2 values of the combined group were significantly lower, and the PaO2 and OI values were higher compared to those of the NIPPV group (P<0.05) (Table-II).

**Table-II:** Comparison of oxygenation between two groups before and after treatment (mmHg).

Item	Group	n	PaCO_2_	PaO_2_	OI
Before treatment	Combined group	74	74.36±6.74	61.32±5.83	193.35±21.33
	NIPPV group	78	75.76±7.08	63.00±5.67	196.34±22.42
	*t*		-1.240	-1.796	-0.843
	*P*		0.217	0.075	0.401
After treatment	Combined group	74	40.46±5.10^a^	83.35±6.74^a^	289.64±27.99^a^
	NIPPV group	78	45.06±5.57^a^	75.81±8.23^a^	269.09±24.46^a^
	*t*		-5.308	6.195	4.826
	*P*		<0.001	<0.001	<0.001

*Note:* Compared to before treatment in the same group,

a *P*<0.05; NIPPV: Noninvasive positive pressure ventilation; PaCO2: arterial partial pressure of carbon dioxide; PaO2: arterial partial pressure of oxygen; OI: oxygenation index.

Before treatment, CAT and SGRQ scores and 6MWT were comparable in the two groups (P>0.05). After treatment, CAT and SGRQ scores of both groups decreased, and 6MWT considerably increased compared to pre-treatment levels. Combined NIPPV and BGF MDI treatment was associated with considerably lower CAT and SGRQ scores and higher 6MWT scores compared to the NIPPV treatment alone (P<0.05) (Table-III). Similarly there was no significant difference in the levels of CRP and IL-6 between the two groups (P>0.05) before treatment,. After treatment, the levels of serum CRP and IL-6 in both groups decreased and were significantly lower in the combined group than in the NIPPV group (P<0.05) (Table-IV).

**Table-III:** Comparison of functional rehabilitation before and after treatment between two groups.

Item	Group	n	CAT (score)	SGRQ (score)	6MWT (m)
Before treatment	Combined group	74	27.58±3.63	65.72±8.67	295.11±43.07
	NIPPV group	78	28.55±3.97	66.83±10.77	298.73±33.96
	*t*		-1.569	-0.706	-0.577
	*P*		0.119	0.484	0.565
After treatment	Combined group	74	12.62±2.87^a^	48.55±6.08^a^	431.80±55.03^a^
	NIPPV group	78	17.04±3.01^a^	53.95±7.70^a^	400.96±49.99^a^
	*t*		-9.252	-4.809	3.619
	*P*		<0.001	<0.001	<0.001

*Note:* Compared to before treatment in the same group,

a *P*<0.05; NIPPV: Noninvasive positive pressure ventilation; CAT: COPD Assessment Test; SGRQ: St George’s Respiratory Questionnaire; 6MWT: 6-minute walking test.

**Table-IV:** Comparison of inflammatory markers between two groups before and after treatment.

Item	Group	n	CRP (mg/L)	IL-6 (ng/L)
Before treatment	Combined group	74	24.38±5.01	25.73±5.89
	NIPPV group	78	25.03±5.90	26.35±6.27
	*t*		-0.727	-0.624
	*P*		0.468	0.534
After treatment	Combined group	74	5.01±1.45^a^	9.31±2.94^a^
	NIPPV group	78	6.43±1.86^a^	11.66±3.09^a^
	*t*		-5.221	-4.795
	*P*		<0.001	<0.001

*Note:* Compared to before treatment in the same group,

a *P*<0.05; NIPPV: Noninvasive positive pressure ventilation; CRP: C-reactive protein; IL-6: interleukin-6.

## DISCUSSION

This study evaluated the efficacy of NIPPV combined with BGF MDI in treating AECOPD. Our results showed that NIPPV combined with BGF MDI treatment in patients with AECOPD was associated with a significant improvement in oxygenation, functional recovery, and inflammatory response after two weeks compared to NIPPV alone. These findings suggest the clinical benefits of using the combination of NIPPV and BGF MDI in treating AECOPD.

Several studies have previously discussed the efficiency of BGF MDI treatment for COPD or AECOPD .^7^,^8^,^13^ Martinez FJ et al.^14^ found that BGF MDI can effectively reduce the mortality rate of patients with COPD. Other studies have confirmed that BGF MDI can effectively improve the lung function of COPD patients and was associated with only minor adverse reactions, such as nasopharyngitis, whose incidence rate is only 8.0%.^8^,^15^ In addition, Nigro SC et al.^8^ showed that patients with COPD treated with BGF MDI showed better improvement in lung function compared to patients treated with budesonide. The results of our study are consistent with the previous research and further confirm the high application value of BGF MDI in COPD.

The results of this study also showed that after treatment, the PaCO2 level in patients who were treated by BGF MDI combined with the NIPPV group was significantly lower compared to the NIPPV alone group. At the same time, PaO2 and OI levels of the combined group were significantly higher. Our results are consistent with the study by Hu et al.^16^ that found that BGF MDI can effectively increase the partial pressure of oxygen in COPD patients and improve their blood gas levels. Together, these results further confirm that NIPPV combined with BGF MDI treatment can further improve patients’ blood oxygen levels and oxygenation efficiency. It is plausible that NIPPV allows COPD patients to breathe more easily and improves their ventilation function by providing respiratory support of positive pressure ventilation. Such support will ultimately improve the oxygen concentration and CO2 concentration in arterial blood gas, alleviate acidosis, ease dyspnea and other symptoms, and reduce CO2 retention, thus promoting the recovery of patients’ respiratory system function.^17^,^18^

Our study showed that after two weeks of the treatment, the CAT and SGRQ scores of the combination group were significantly lower than those of the NIPPV group, while the 6MWT level was significantly higher than that of the NIPPV group. We may speculate that the observed effect is mainly due to the components of the BGF MDI. Budesonide has a strong anti-inflammatory effect, which may reduce the release of active mediators such as histamine, thereby alleviating airway inflammation and relieving symptoms such as breathing difficulties. Glycopyrrolate, as an anticholinergic drug, can effectively reduce vagal tone, dilate bronchial smooth muscle, and improve ventilation, and formoterol fumarate has a strong and sustained bronchodilator effect, which helps reduce airway spasms and alleviate patient symptoms.^7^–^9^,^13^,^19^,^20^ A study by Martinez FJ et al.^13^ that included 8509 patients showed that compared to glycopyrrolate/formoterol fumarate and budesonide/formoterol fumarate, budesonide/glycopyrrolate/formoterol fumarate treatment was more effective in improving the total SGRQ score of COPD patients. The research results of Wang C et al.^20^ also indicate that budesonide/glycopyrrolate/fumarate formoterol can reduce the incidence of exacerbation in COPD patients and have good tolerability, which is consistent with our observations.

In this study, the levels of CRP and IL-6 in the post-treatment combination group were lower than those in the NIPPV group alone. Inflammatory response plays an important role in the onset and progression of COPD, and the interaction between inflammatory response and immunity can promote the progression of COPD.^21^ CRP is an important sensitive biomarker that reflects the degree of inflammation in the body. Under normal physiological conditions, the expression level of CRP is low; however, it dramatically increases in case of inflammation.^21^,^22^ IL-6 also participates in multiple inflammatory and immune responses in the body, with various functions, and can interact with multiple markers to mediate immune regulation.^23^

Our results further confirm that NIPPV combined with BGF MDI can effectively downregulate the levels of inflammatory markers in COPD patients, which is beneficial for reducing the inflammatory response. Our results are consistent with those of Jiang DH et al.^24^, Lin YH et al.^25^ and Mei X et al.^26^ We suggest that this inhibition of the inflammatory response is mediated by the combination of the anti-inflammatory effect of budesonide, and the broncho-dilating effect of glycopyrrolate and formoterol fumarate. The synergistic action of these agents improves airway spasms, reduces mucus secretion, restores normal ventilation function, and improves airway mucus hypersecretion status.^24^,^25^ In summary, this study confirms that compared to NIPPV alone, NIPPV combined with BGF MDI can achieve higher benefits in AECOPD patients in the short term. This study also supplements the limited literature on the treatment of AECOPD patients with NIPPV combined with BGF MDI. This provides advice for clinical treatment. However, long-term follow-up studies are needed to confirm the impact of this treatment method on patients’ long-term function and quality of life.

### Limitations:

Firstly, it is a single-center retrospective analysis with a small sample size, which may limit the generalization of the findings. Secondly, subgroup analysis was not conducted on patients based on their COPD condition. Future studies should verify the benefits of the combined treatment in patients with AECOPD. Thirdly, in this study, patients who completed two weeks of treatment without diagnosed disease progression were allowed to continue using BGF MDI and were deemed to still benefit from the study treatment based on the researcher’s assessment. Finally, a longer follow-up period is needed to verify the long-term effects of NIPPV combined with BGF MDI on AECOPD patients’ function and quality of life.

## CONCLUSION

Compared with NIPPV alone, the combination of NIPPV and BGF MDI can effectively restore oxygenation, improve functional status, alleviate clinical symptoms, and reduce the degree of inflammatory response in COPD patients. This method of treatment may be recommended for use in clinical practice to improve the status of patients with COPD.

### Authors’ contributions:

**JS:** Study design, literature search and manuscript writing.

**XX:** Data collection, data analysis and interpretation. Critical Review

**JS:** Manuscript revision and validation, Critical analysis.

All authors have read, approved the final manuscript and are responsible for the integrity of the study.
